# Integrative Analysis of Gene Expression Through One-Class Logistic Regression Machine Learning Identifies Stemness Features in Multiple Myeloma

**DOI:** 10.3389/fgene.2021.666561

**Published:** 2021-08-16

**Authors:** Chunmei Ban, Feiyan Yang, Min Wei, Qin Liu, Jiankun Wang, Lei Chen, Liuting Lu, Dongmei Xie, Lie Liu, Jinxiong Huang

**Affiliations:** Department of Hematology, Liuzhou People’s Hospital, Liuzhou, China

**Keywords:** stemness index, mRNAsi, multiple myeloma, OCLR, prognosis, WGCNA

## Abstract

Tumor progression includes the obtainment of progenitor and stem cell-like features and the gradual loss of a differentiated phenotype. Stemness was defined as the potential for differentiation and self-renewal from the cell of origin. Previous studies have confirmed the effective application of stemness in a number of malignancies. However, the mechanisms underlying the growth and maintenance of multiple myeloma (MM) stem cells remain unclear. We calculated the stemness index for samples of MM by utilizing a novel one-class logistic regression (OCLR) machine learning algorithm and found that mRNA expression-based stemness index (mRNAsi) was an independent prognostic factor of MM. Based on the same cutoff value, mRNAsi could stratify MM patients into low and high groups with different outcomes. We identified 127 stemness-related signatures using weighted gene co-expression network analysis (WGCNA) and differential expression analysis. Functional annotation and pathway enrichment analysis indicated that these genes were mainly involved in the cell cycle, cell differentiation, and DNA replication and repair. Using the molecular complex detection (MCODE) algorithm, we identified 34 pivotal signatures. Meanwhile, we conducted unsupervised clustering and classified the MM cohorts into three MM stemness (MMS) clusters with distinct prognoses. Samples in MMS-cluster3 possessed the highest stemness fractions and the worst prognosis. Additionally, we applied the ESTIMATE algorithm to infer differential immune infiltration among the three MMS clusters. The immune core and stromal score were significantly lower in MMS-cluster3 than in the other clusters, supporting the negative relation between stemness and anticancer immunity. Finally, we proposed a prognostic nomogram that allows for individualized assessment of the 3- and 5-year overall survival (OS) probabilities among patients with MM. Our study comprehensively assessed the MM stemness index based on large cohorts and built a 34-gene based classifier for predicting prognosis and potential strategies for stemness treatment.

## Introduction

Multiple myeloma (MM), a clonal B-cell malignancy (Bataille and Harousseau, 1997), is characterized by the aberrant proliferation of bone marrow plasma cells and the overproduction of light-chain or monoclonal immunoglobulin (Röllig et al., 2015; Dhakal et al., 2016). Among the diseases with abnormal plasma cells, MM is the second most common hematologic malignancy (Röllig et al., 2015). Owing to the development of immunomodulators and proteasome inhibitors, patients with MM have significantly improved survival over the past decade. However, progressive disease remains a common cause of fatal outcomes (Martinez-Lopez et al., 2011). As a result, it is essential to understand the underlying mechanisms that contribute to disease relapse and progression in MM, as well as novel targets for therapeutic improvements or prognostic prediction.

Stemness was defined as the potential for differentiation and self-renewal from the cell of origin (Seguin et al., 2015; Prasetyanti and Medema, 2017). It has been reported that the gradual loss of differentiation capacity and acquisition of stem cell-like characteristics are important factors that promote tumor progression (Seguin et al., 2015). A growing body of research has confirmed the existence of cancer stem cells in multiple tumors, including hematological malignancies (Lapidot et al., 1994; Singh et al., 2004; O’Brien et al., 2007). Cancer stem cells play a key role in tumorigenesis, progression, metastasis, and drug resistance. Therapies targeting cancer stem cells are of great value for tumor prevention and treatment. For example, the use of retinoic acid to induce the maturation and differentiation of malignant proliferating cells has achieved great success in the clinical treatment of promyelocytic leukemia (Nowak et al., 2009). With the application of serial transplantation models and clonogenic *in vitro* assays, MM stem cells have been suggested to be part of a subset of *CD38^–^CD19^+^CD27^+^* B-cell precursors that do not express the classic MM markers *CD38* or CD138 (Matsui et al., 2008). These cells with clonogenic potential could mediate tumor regrowth and chemoresistance. The one-class logistic regression (OCLR) machine learning algorithm is an effective method of quantifying the cancer stemness index using two independent indices (Malta et al., 2018; Lian et al., 2019). One is the stemness index [mRNA expression-based stemness index (mRNAsi)] based on gene expression that reflects mRNA expression, and the other is mDNAsi, which reflects epigenetic characteristics (Sokolov et al., 2016). Previous studies have proven the effective application of stemness indices calculated by the OCLR algorithm in a variety of malignant tumors. However, there are few studies on the role and prognostic value of MM stemness (MMS); hence, it is an urgent need to develop prognostic or predictive biomarkers associated with stemness index.

In this study, we collected a total of 1,095 newly diagnosed or pre-treatment MM patients with expression data and clinical information and systematically evaluated the MM stemness index (mRNAsi) using the OCLR algorithm. By combining weighted gene co-expression network analysis (WGCNA) with MM mRNAsi, we searched for key genes related to stemness in 1,095 MM patients. The analysis of gene and module functions showed significance in MM. We classified MM patients into different subgroups based on the expression of stemness-related genes. Finally, mRNAsi was integrated with other clinicopathological characteristics to construct a nomogram to predict the prognosis of patients with MM.

## Materials and Methods

### Data Collection and Pre-processing

**FIGURE 1:**
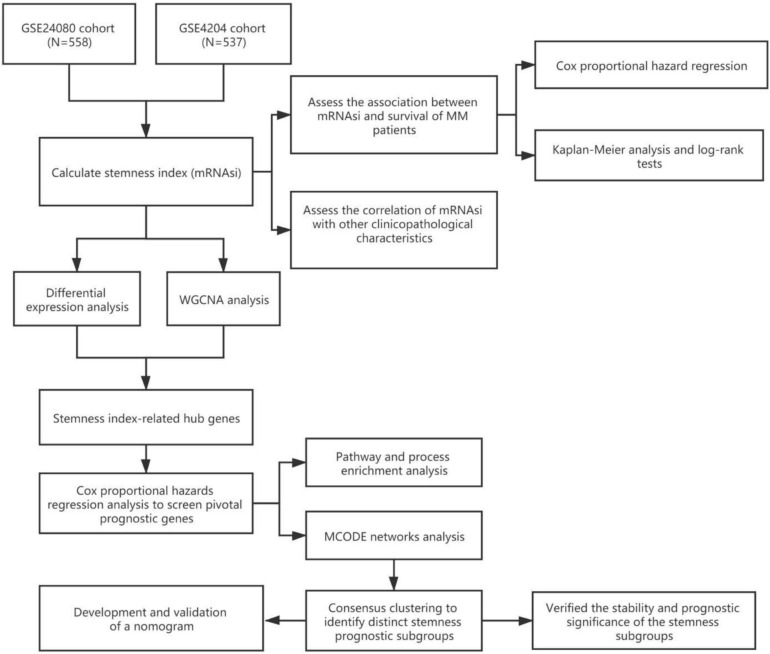
Workflow of this research.

The workflow of this study is shown in Figure 1. In this study, two MM cohorts were downloaded from the Gene Expression Omnibus (GEO)^1^ database. The related information contained clinical, molecular, and microarray datasets. Samples in the GSE4204 cohort (*N* = 538) were pre-treated with bone marrow aspirates from MM patients (Driscoll et al., 2010). The GSE24080 cohort consisted of 559 newly diagnosed MM patients (Shi et al., 2010).

The gene expression values in the two cohorts were analyzed using the robust multi-array average algorithm for background correction, quantile normalization, and final summarization. After excluding samples without survival information, a total of 1,095 samples were selected for subsequent analysis (558 cases from the GSE24080 cohort and 537 cases from the GSE4204 cohort).

### Calculation and Prognosis Evaluation of mRNAsi

Malta et al. (2018) provided a novel OCLR machine learning algorithm to assess oncogenic dedifferentiation that considered mRNAsi. We utilized the method to calculate the mRNAsi for each MM patient. The gene expression-based stemness index was mapped from zero to one. We assessed the prognostic value of mRNAsi and its relationship with other clinical features in different cohorts. First, we utilized univariate and multivariable Cox proportional hazard regression to calculate the hazard ratios (HRs) for overall survival (OS). Second, each cohort of MM patients was divided into low and high mRNAsi groups based on the same cutoff value of mRNAsi (0.4124166, identified by the *Survminer* package), and the Kaplan–Meier method and log-rank tests were used to determine the significance of survival differences.

### Identification of Key Modules and Genes Associated With mRNAsi by WGCNA

The WGCNA was developed to discover correlations among genes by constructing significant modules (Langfelder and Horvath, 2008). The mRNAsi and expression modules were calculated using WGCNA to identify key modules and genes associated with mRNAsi. Module eigengenes (MEs) were defined as the first principal component of each gene module and were adopted as the representative of all genes in each module. Gene significance (GS), as the mediator *p*-value (GS = lgP) for each gene, represented the degree of linear correlation between gene expression of module and mRNAsi or other clinical features. MRNAsi-related modules were defined according to *P* < 0.01, and a higher GS value was used for further analysis. Finally, we selected the intersection of the key genes identified in different data sets.

### Screened Differentially Expressed Genes

The *limma* R package was used to screen differentially expressed genes (DEGs) between the high and low mRNAsi groups (Ritchie et al., 2015). The selection criteria were as follows: adjusted *P*-value < 0.0001 and | log2 fold change| > 0.5.

### Functional Annotation and Pathway Enrichment Analysis

We first selected the intersection between the modular genes identified by WGCNA and the DEGs identified by differential expression analysis and then adopted a univariate Cox regression model to assess the prognostic significance of the overlapping genes. Only genes with a *P*-value < 0.05 were considered as prognosis-related hub genes. Functional annotation and pathway enrichment analysis of these hub genes was performed using Metascape (Zhou et al., 2019).^2^

### Consensus Clustering and Prognostic Analysis Based on mRNAsi-Related Hub Genes

Protein-protein interaction enrichment analysis was conducted, and the molecular complex detection (MCODE) algorithm (Bader and Hogue, 2003) was used to identify densely connected network components. Based on the 34 mRNAsi-related hub genes identified by the MCODE algorithm, we subsequently performed unsupervised clustering on MM patients. The R package *ConsensusClusterPlus* was used to conduct unsupervised clustering (1,000 iterations, 80% resampling), and k-means and Euclidean distances were used as the clustering algorithm and distance metric, respectively (Wilkerson and Hayes, 2010). We then assessed the prognosis in each MMS-cluster via Kaplan–Meier analysis. Meanwhile, mRNAsi was also compared in distinct MMS clusters.

### Development and Validation of a Nomogram for Prognosis Prediction of MM Patients

To develop a clinically applicable method of predicting MM prognosis, we integrated mRNAsi and other clinicopathological covariates to build a nomogram. Predictive factors included mRNAsi, cytogenetic abnormalities, albumin (ALB), beta-2 microglobulin (B2M), and lactate dehydrogenase (LDH). The nomogram was verified using receiver operating characteristic (ROC) curves and calibration curves.

### Statistical Analyses

The Wilcoxon rank-sum test was used to compare two groups with non-normally distributed variables, while Student’s *t*-test was conducted to compare groups with normally distributed variables. The one-way analysis of variance or Kruskal–Wallis tests were used to compare differences between three or more groups. Based on the correlation between mRNAsi and patient survival, the cutoff point of mRNAsi was determined using the *Survminer* R package. The “surv-cutpoint” function, which repeatedly tested all potential cut points to find the maximum rank statistic, was applied to dichotomize mRNAsi, and the patients were then divided into high and low mRNAsi groups based on the maximally selected log-rank statistics to decrease the batch effect of calculation. The survival curves for the prognostic analysis were generated using the Kaplan–Meier method, and log-rank tests were used to determine the significance of differences. Independent prognostic factors were ascertained using the multivariable Cox regression model.

## Results

### The Association Between mRNAsi and Patient Survival

**FIGURE 2:**
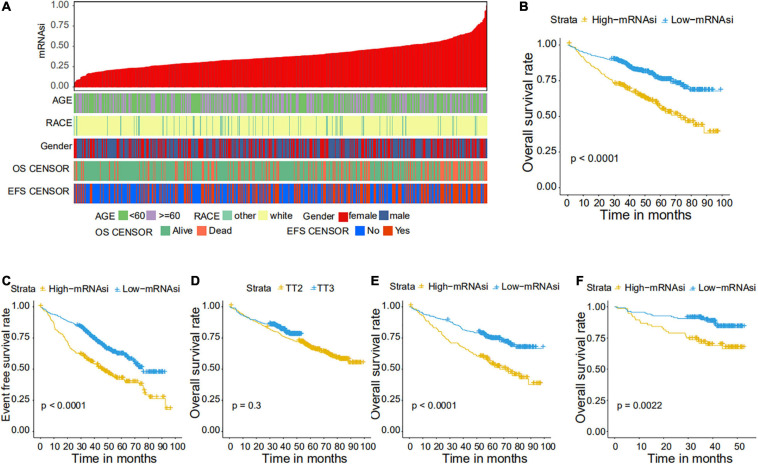
Clinical characteristics related to the mRNA expression-based stemness index (mRNAsi) in the GSE24080 cohort. **(A)** Overview of the association between demographic characteristics and mRNAsi in MM. Columns represent samples sorted by mRNAsi from low to high (top row), and rows represent the demographic factors associated with mRNAsi. **(B,C)** The Kaplan–Meier curve was used to determine the overall survival (OS) **(B)** and event-free survival (EFS) **(C)** of patients in the multiple myeloma (MM) group with high and low mRNAsi. **(D)** The Kaplan–Meier curve was used to determine the OS of patients treated with the TT2 or TT3 regimen. **(E)** The Kaplan–Meier curve was used to determine the OS of high and low mRNAsi patients treated with the TT2 regimen. **(F)** The Kaplan–Meier curve was used to determine the OS of high and low mRNAsi patients treated with the TT3 regimen.

**FIGURE 3:**
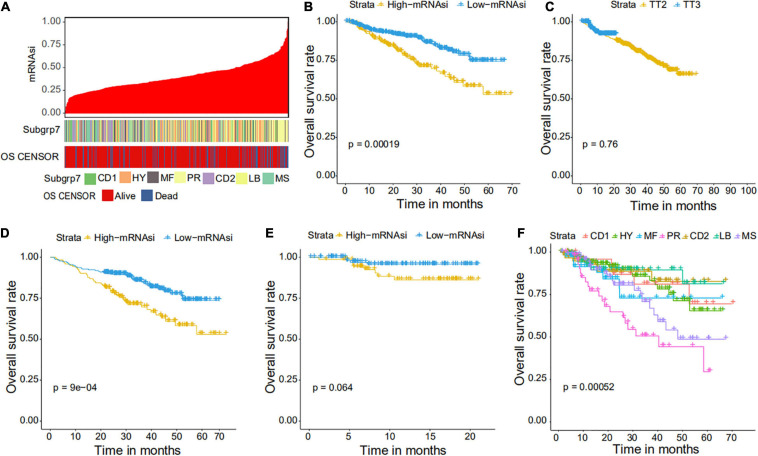
Clinical characteristics related to the mRNAsi in the GSE4204 cohort. **(A)** Overview of the association between subtype and mRNAsi in MM. The columns represent samples sorted by mRNAsi from low to high (top row), while the rows represent the demographic factors associated with mRNAsi. **(B)** The Kaplan–Meier curve was used to determine the OS of patients in the MM group with high and low mRNAsi. **(C)** The Kaplan–Meier curve was used to determine the OS of patients treated with the TT2 or TT3 regimen. **(D)** The Kaplan–Meier curve was used to determine the OS of high and low mRNAsi patients treated with the TT2 regimen. **(E)** The Kaplan–Meier curve was used to determine the OS of high and low mRNAsi patients treated with the TT3 regimen. **(F)** The Kaplan–Meier curve was used to determine the OS of patients of different MM subtypes.

We collected 1,095 MM samples with clinical information and corresponding expression data to systematically characterize the stemness features of MM. The overall characteristics of the MM cohort are listed in Table 1. MM samples were sorted according to their mRNAsi values (from low to high stemness index) and examined whether any clinical characteristic/molecular/demographic was associated with either a low or high stemness index (Figures 2A, 3A). We did not find significant differences in mRNAsi in terms of gender and race, but we found that patients who died and patients who experienced adverse events occurred often showed a higher mRNAsi.

**TABLE 1:** Baseline characteristics of multiple myeloma (MM) patients.

**Characteristic**	**Overall**	**GSE24080**	**GSE4204**
Project (%)	1,095	558	537
GSE24080	558 (51.0)	558 (100.0)	0 (0.0)
GSE4204	537 (49.0)	0 (0.0)	537 (100.0)
**OS CENSOR (%)**			
Alive	832 (76.0)	387 (69.4)	445 (82.9)
Dead	263 (24.0)	171 (30.6)	92 (17.1)
OS TIME (median [IQR])	37.10 [19.06,53.55]	48.23 [34.63,64.07]	23.97 [12.07,39.67]
**Treatment (%)**			
TT2	688 (62.8)	344 (61.6)	344 (64.1)
TT3	407 (37.2)	214 (38.4)	193 (35.9)
SUBGRP7 (%)	127 (23.6)	0 (NaN)	127 (23.6)
CD1	28 (5.2)	0 (NaN)	28 (5.2)
CD2	59 (11.0)	0 (NaN)	59 (11.0)
HY	114 (21.2)	0 (NaN)	114 (21.2)
LB	58 (10.8)	0 (NaN)	58 (10.8)
MF	37 (6.9)	0 (NaN)	37 (6.9)
MS	67 (12.5)	0 (NaN)	67 (12.5)
PR	47 (8.8)	0 (NaN)	47 (8.8)
**AGE (%)**			
<60	317 (56.8)	317 (56.8)	0 (NaN)
≥60	241 (43.2)	241 (43.2)	0 (NaN)
**Gender (%)**			
Female	222 (39.8)	222 (39.8)	0 (NaN)
Male	336 (60.2)	336 (60.2)	0 (NaN)
**RACE (%)**			
White	496 (88.9)	496 (88.9)	0 (NaN)
Other	62 (11.1)	62 (11.1)	0 (NaN)
**EFS CENSOR (%)**			
No	310 (55.6)	310 (55.6)	0 (NaN)
Yes	248 (44.4)	248 (44.4)	0 (NaN)
EFS TIME (median [IQR])	42.40 [29.23,56.66]	42.40 [29.23,56.66]	NA [NA, NA]
ISOTYPE (%)	15 (2.7)	15 (2.7)	0 (NaN)
FLC	84 (15.1)	84 (15.1)	0 (NaN)
IgA	133 (23.8)	133 (23.8)	0 (NaN)
IgD	3 (0.5)	3 (0.5)	0 (NaN)
IgG	312 (55.9)	312 (55.9)	0 (NaN)
Non-secretory	6 (1.1)	6 (1.1)	0 (NaN)
NSE	2 (0.4)	2 (0.4)	0 (NaN)
NA	3 (0.5)	3 (0.5)	0 (NaN)
B2M (%)	1 (0.2)	1 (0.2)	0 (NaN)
<3.5	319 (57.2)	319 (57.2)	0 (NaN)
≥5.5	118 (21.1)	118 (21.1)	0 (NaN)
3.5-5.5	120 (21.5)	120 (21.5)	0 (NaN)
CRP (median [IQR])	4.40 [1.20, 11.00]	4.40 [1.20, 11.00]	NA [NA, NA]
CREAT (median [IQR])	1.00 [0.80, 1.20]	1.00 [0.80, 1.20]	NA [NA, NA]
LDH (median [IQR])	156.50 [127.00, 199.00]	156.50 [127.00, 199.00]	NA [NA, NA]
ALB (median [IQR])	4.10 [3.70, 4.40]	4.10 [3.70, 4.40]	NA [NA, NA]
HGB (median [IQR])	11.30 [9.80, 12.60]	11.30 [9.80, 12.60]	NA [NA, NA]
ASPC (median [IQR])	40.00 [23.00, 60.00]	40.00 [23.00, 60.00]	NA [NA, NA]
BMPC (median [IQR])	45.00 [21.25, 70.00]	45.00 [21.25, 70.00]	NA [NA, NA]
MRI (median [IQR])	5.00 [0.00, 16.00]	5.00 [0.00, 16.00]	NA [NA, NA]
Cyto_Abn (%)			
No	352 (63.1)	352 (63.1)	0 (NaN)
Yes	206 (36.9)	206 (36.9)	0 (NaN)

*OS CENSOR, (dead = event) overall survival censor (dead); EFS CENSOR, (yes = event) event-free survival censor (disease relapse or progression); AGE, age at registration (years); B2M, beta-2 microglobulin (mg/l); CRP, C-reactive protein (mg/l); CREAT, creatinine (mg/dl); LDH, lactate dehydrogenase (U/l); ALB, albumin (35 g/l); HGB, hemoglobin (g/dl); ASPC, aspirate plasma cells (%); BMPC, bone marrow biopsy plasma cells (%); MRI, number of magnetic resonance imaging (MRI)-defined focal lesions (skull, spine, pelvis). Cytogenetic abnormality an indicator of the detection of cytogenetic abnormalities. Yes = abnormalities were detected, No = were not detected or were absent.*

### Correlations of mRNAsi With Clinical Prognosis in MM

**FIGURE 4:**
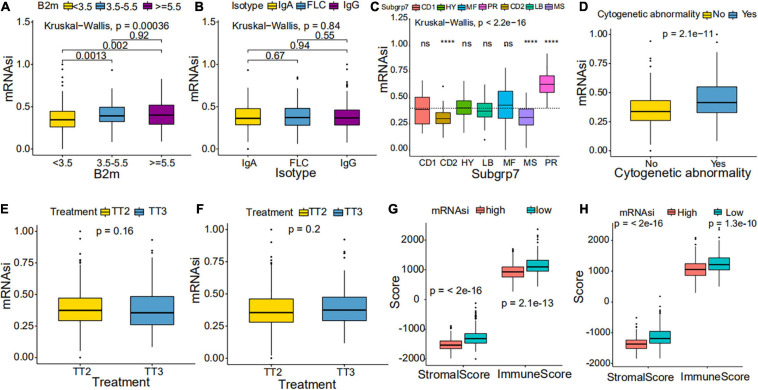
Clinical characteristics in low mRNAsi and high mRNAsi MM patients. **(A,B)** Differences in mRNAsi between distinct B2M groups **(A)** and isotype groups **(B)** in patients with MM. The upper and lower ends of the boxes represent the interquartile range of values. The lines in the boxes represent median values. **(C,D)** Difference in mRNAsi between distinct subgroups **(C)** and cytogenetic abnormalities groups **(D)** in patients with MM. **(E,F)** Difference in mRNAsi between distinct treatment regimen groups in patients of the GSE24080 cohort **(E)** or the GSE4204 cohort **(F)**. **(G,H)** Difference in stromalscore and immunescore between high and low mRNAsi samples of the GSE24080 cohort **(G)** or the GSE4204 cohort **(H)**. ****p < 0.0001.

We further explored the prognostic value of mRNAsi in patients with MM. First, the multivariate Cox regression model analysis in different cohorts confirmed mRNAsi as an independent and robust prognostic biomarker for evaluating MM patient outcomes (Table 2 and Supplementary Tables 1, 2). Second, based on the cutoff point (0.4124166) identified by the *Survminer* R package, each MM cohort was separated into low and high mRNAsi groups. The Kaplan–Meier analysis confirmed that the high-mRNAsi group had shorter OS and event-free survival (EFS) than the low mRNAsi group in the GSE24080 cohort (Figures 2B,C). Consistent with this, in the GSE4204 cohort, the Kaplan–Meier analysis indicated that the high-mRNAsi group had shorter OS than the low mRNAsi group (Figure 3B). Surprisingly, we found that in both the GSE24080 and GSE4204 cohorts, the treatment regimen (TT2 or TT3) did not improve the survival of MM patients (Figures 2D, 3C). Regardless of the treatment plan (TT2 or TT3), it failed to improve the survival disadvantages of patients in the high-mRNAsi group (Figures 2E,F, 3D,E). Consistently with previous reports (Zhan et al., 2006), our analysis found that among the seven subgroups of MM, the PR subgroup had the worst prognosis, followed by the MS and MF subgroups (Figure 3F). Finally, we investigated the association between mRNAsi and other clinical characteristics. We found that the difference in mRNAsi among the groups was statistically significant when comparing groups of patients with B2M ≥ 3.5 and those with B2M < 3.5 (Figure 4A). There was no significant difference in mRNAsi between the different isotype groups (Figure 4B). However, for the seven subgroups of MM, consistent with poor survival, patients in the PR subgroups possessed the highest mRNAsi (Figure 4C). In terms of cytogenetic abnormalities, patients with cytogenetic abnormalities tended to have higher mRNA levels (Figure 4D). Since different therapies (TT2 or TT3) failed to improve the survival of MM patients, we further investigated whether there were any differences in mRNAsi among MM patients who received different therapies. Consistent with our conjecture, different treatment regimens failed to effectively affect mRNAsi (Figures 4E,F). Based on the ESTIMATE algorithm, we calculated the individual stromal and immune scores to evaluate the level of infiltrating stromal and immune cells in any given MM sample (Yoshihara et al., 2013). We found that the high-mRNAsi group had lower immune and stromal scores than the low mRNAsi group, which suggested that MM patients with high mRNAsi had a lower level of infiltration of tumor microenvironment (TME) cells (Figures 4G,H).

**TABLE 2:** Univariate and multivariable cox regression analysis of clinical features and OS in the GSE24080 cohort of MM patients.

	**Univariate Cox regression analysis**		**Multivariable Cox regression analysis**	
	**HR (95%CI)**	***p* value**	**HR (95%CI)**	***p* value**
Treatment	0.82431 (0.57344–1.1849)	0.29671	0.85959 (0.56569–1.3062)	0.4785
Age	1.4345 (1.0616–1.9385)	0.018833	1.2299 (0.88208–1.715)	0.22238
Gender	0.95881 (0.70659–1.301)	0.78707	1.1181 (0.7849–1.5929)	0.53626
Race	1.0527 (0.65329–1.6965)	0.83276	0.98993 (0.58153–1.6851)	0.97025
B2M	1.0834 (1.0647–1.1025)	2.21E-19	1.1043 (1.0627–1.1475)	4.20E-07
CRP	1.0032 (0.99834–1.0081)	0.19614	0.99659 (0.98718–1.0061)	0.47955
CREAT	1.2329 (1.1382–1.3354)	2.79E-07	0.92317 (0.79575–1.071)	0.29151
LDH	1.0062 (1.0046–1.0079)	3.84E-13	1.0044 (1.0022–1.0065)	5.47E-05
ALB	0.58079 (0.471–0.71618)	3.73E-07	0.73108 (0.54743–0.97634)	0.033821
HGB	0.87091 (0.80301–0.94456)	0.00084631	1.0076 (0.90369–1.1235)	0.89144
ASPC	1.0102 (1.004–1.0164)	0.0011275	0.99881 (0.98875–1.009)	0.81831
BMPC	1.0096 (1.0038–1.0155)	0.0012532	1.0003 (0.99088–1.0099)	0.94347
MRI	1.0172 (1.0083–1.0261)	0.00012887	1.0089 (0.99865–1.0193)	0.088955
Cyto_Abn	2.2708 (1.6805–3.0685)	9.32E-08	1.7924 (1.2669–2.5358)	0.00098068
mRNAsi	28.219 (10.696–74.45)	1.50E-11	5.0703 (1.3013–19.756)	0.019312
StromalScore	0.9994 (0.99881–0.99998)	0.04337	1.0003 (0.99956–1.0011)	0.39522
ImmuneScore	0.99958 (0.99906–1.0001)	0.12077	1.0002 (0.99946–1.0009)	0.64608

### Weighted Gene Co-expression Network Analysis: Identification of the Most Significant Modules and Genes

**FIGURE 5:**
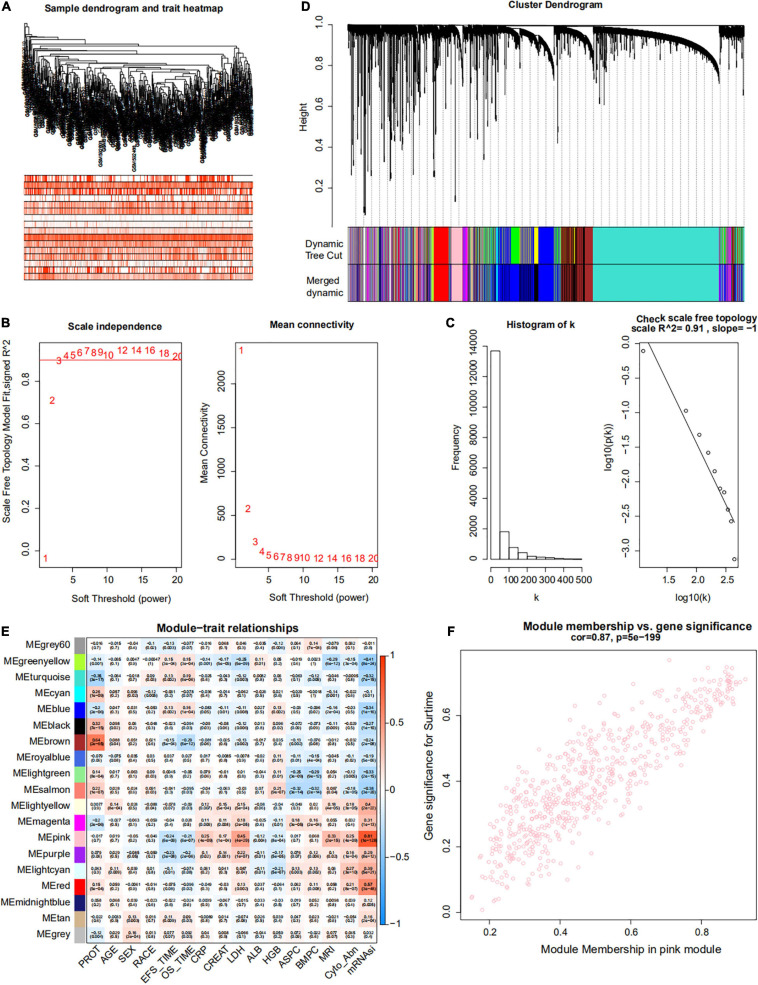
Weighted gene co-expression network of MM of the GSE24080 cohort. **(A)** Sample dendrogram and trait heatmap. The colors represent the proportion of clinical traits. **(B)** Left panel: the scale-free topology fit index for soft-thresholding powers. Right panel: mean connectivity for soft-thresholding powers. **(C)** Scale-free *R*2 (*R*2 = 0.91). **(D)** Clustering dendrogram of genes in patients with MM. **(E)** Pertinence between clinical traits and gene modules. **(F)** Scatter plots of gene significance (GS) for mRNAsi corresponding to module membership in the pink module, with their correlation coefficients and *P*-values.

**FIGURE 6:**
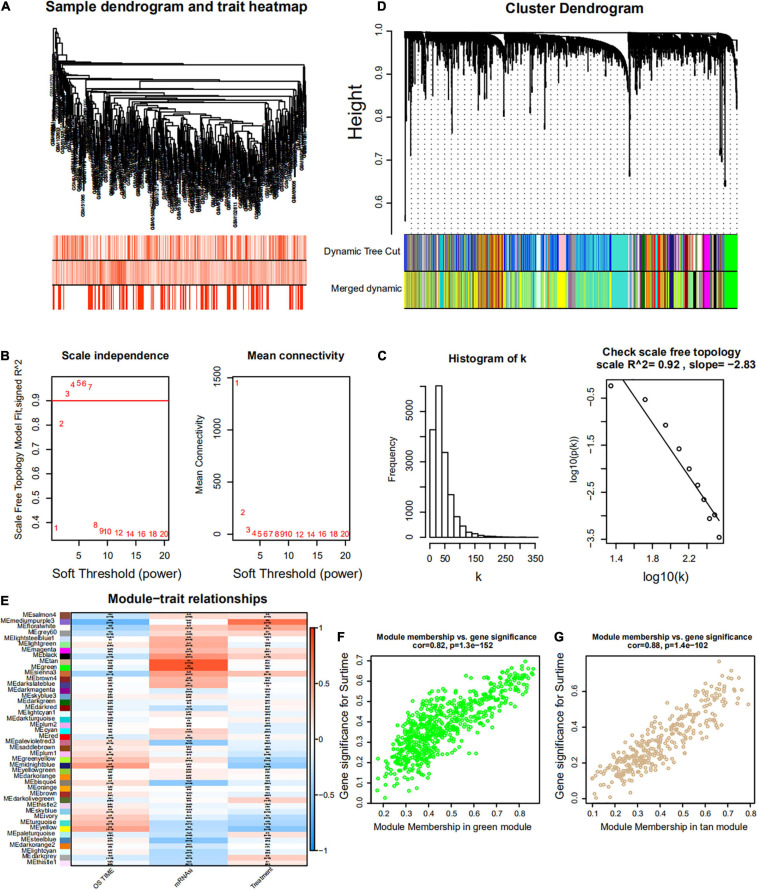
Weighted gene co-expression network of MM of the GSE4204 cohort. **(A)** Sample dendrogram and trait heatmap. The colors represent the proportion to clinical traits. **(B)** Left panel: the scale-free topology fit index for soft-thresholding powers. Right panel: mean connectivity for soft-thresholding powers. **(C)** Scale-free *R*2 (*R*2 = 0.92). **(D)** Clustering dendrogram of gene in patients with MM. **(E)** Pertinence between clinical traits and gene modules. **(F,G)** Scatter plots of GS for mRNAsi corresponding to module membership in green and tan modules, with their correlation coefficients and *P*-values.

**FIGURE 7:**
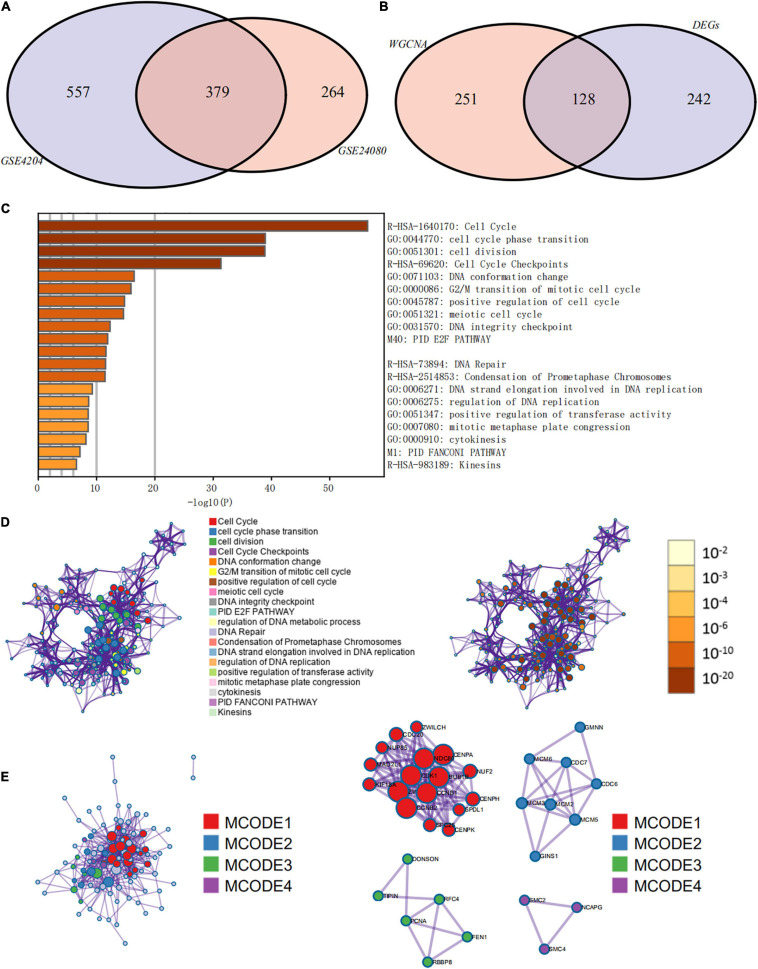
Functional annotation and pathway enrichment analysis of key prognostic genes that were identified by weighted gene co-expression network analysis (WGCNA) and differential expression analysis. **(A)** Venn diagram of genes identified by WGCNA in the GSE24080 and GSE4204 cohorts. **(B)** Venn diagram of genes identified by WGCNA and differential expression analysis. **(C)** Bar graph of enriched terms of stemness-related genes, colored by *P*-values. **(D)** Network of enriched terms: (left panel) colored by cluster ID, where nodes that share the same cluster ID are typically close to each other; (right panel) colored by *P*-value, where terms containing more genes tend to have more significant *P*-values. **(E)** Protein–protein interaction network and molecular complex detection components identified in the gene lists of the 127 stemness-related signatures.

Weighted gene co-expression network analysis was used to build a gene co-expression network to classify all genes into biological gene modules based on average linkage hierarchical clustering and further identify genes strongly associated with MM stemness (Figures 5A,D, 6A,D). The soft-thresholding powers in the WGCNA were determined based on scale-free R2 (Figures 5B,C, 6B,C). The pink module had the highest correlation with mRNAsi in the GSE24080 cohort (Figures 5E,F). Additionally, the tan and green modules were highly associated with mRNAsi in the GSE4204 cohort (Figures 6E–G). The pink module contained 643 genes (Figure 5F), and tan and green contained 313 and 623 genes, respectively (Figures 6F,G). There were 379 overlapping genes in the three modules (Figure 7A). A total of 379 overlapping genes were retained for further analysis.

### Differentially Expressed Genes Between Low and High mRNAsi Groups of MM Patients

Since the prognosis of MM patients in the high and low mRNAsi groups differed significantly, we conducted a differential expression analysis between high and low mRNAsi groups to identify the differentially expressed key genes that regulate stemness. We identified 370 DEGs, of which 326 were upregulated and 44 were downregulated in the high-mRNAsi group. After merging the 370 DEGs with the 379 co-expressed genes identified by WGCNA, 128 hub genes were retained for further analysis (Figure 7B).

### Pathway and Process Enrichment Analysis of Hub Prognostic Genes

The prognostic evaluation of the 128 hub genes was performed using the Cox proportional hazards regression analysis in the GSE24080 cohort dataset. In total, 127 hub genes were identified as significantly prognostic of the outcome (Supplementary Table 3, *P* < 0.05). Functional enrichment analysis was performed using Metascape to elucidate the biological functions of the 127 hub prognostic genes (Figure 7C). The enrichment analysis suggested that these genes were significantly enriched in pathways and processes related to cell cycle, cell differentiation, and DNA replication and repair (Figure 7D, hypergeometric test *P* < 0.01). Finally, based on the MCODE algorithm, the 127 hub prognostic genes were divided into four densely connected network components. The MCODE networks identified for the individual gene lists are shown in Figure 7E. A total of four major gene modules (MCODE 1–4) were identified, including 34 hub prognostic genes (Supplementary Table 4).

### Consensus Clustering to Distinguish Different Stemness Prognostic Subtypes

**FIGURE 8:**
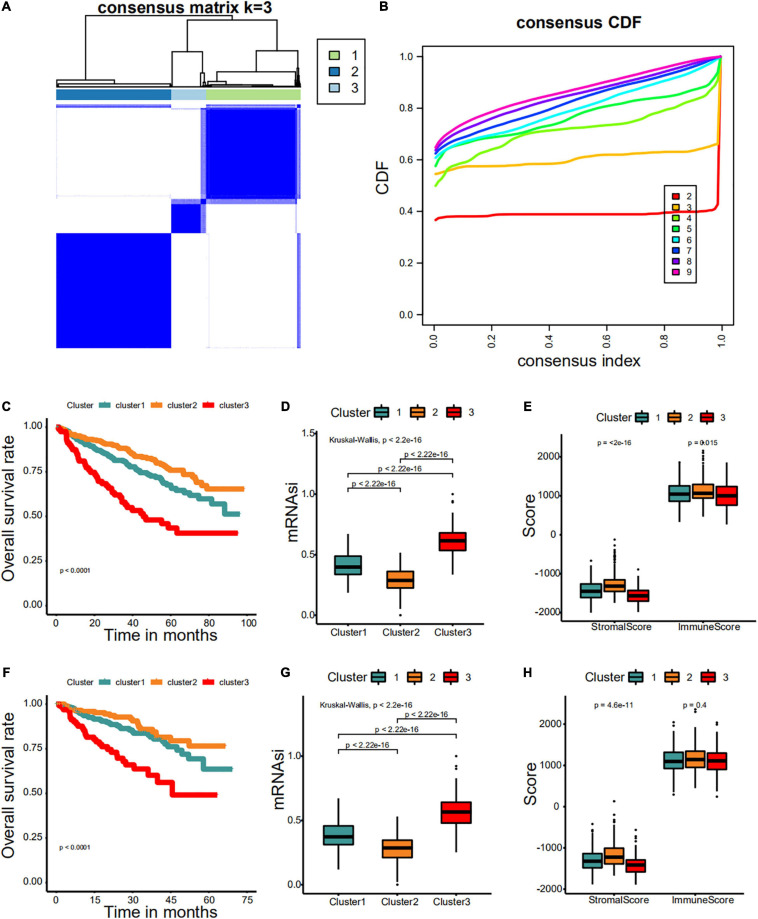
Consensus clustering identified distinct multiple myeloma stemness (MMS) clusters with different prognoses. **(A)** Consensus score matrix for MM samples when *k* = 3. **(B)** The cumulative distribution function (CDF) describes a real random variable of its probability distribution based on consensus scores for different subtype numbers (*k* = 2–9). **(C)** The Kaplan–Meier curve was used to determine the OS of patients of different MMS clusters in the GSE24080 cohort. **(D)** Difference in mRNAsi among distinct MMS clusters in patients of the GSE24080 cohort. **(E)** Difference in stromalscore and immunescore among distinct MMS clusters in patients of the GSE24080 cohort. **(F)** The Kaplan–Meier curve was used to determine the OS of patients of different MMS clusters in the GSE4204 cohort. **(G)** Difference in mRNAsi among distinct MMS clusters in patients of the GSE4204 cohort. **(H)** Difference in stromalscore and immunescore among distinct MMS clusters in patients of the GSE4204 cohort.

First, we identified different stemness prognostic subgroups in the GSE24080 cohort. We utilized the 34 stemness-related signatures of MCODE 1–4 to conduct unsupervised clustering and identified the stemness molecular subtypes of MM for prognostic analysis. The R package of *ConsensusClusterPlus* was used to iterate 1,000 times for the stabilization of classification categories (parameters: pItem = 0.8, reps = 1,000, pFeature = 1), and three distinct stemness molecular subgroups were eventually identified using unsupervised clustering (Figures 8A,B). Unsupervised clustering is a useful technique in tumor research, where intrinsic groups sharing biological characteristics may exist but are unknown. A Kaplan–Meier analysis was conducted across the three clusters, where MMS-cluster3 had the worst OS prognosis and MMS-cluster2 had the most favorable prognosis (Figure 8C, log-rank test *P* < 0.0001). In agreement with these results, MMS-cluster3 had the highest mRNAsi, and MMS-cluster2 had the lowest mRNAsi (Figure 8D). In addition, to verify our stemness prognostic subgroups, based on the 34 stemness-related signatures, we also performed unsupervised clustering and prognostic analysis on the GSE4204 cohort. Consistent with the above results, the MM samples could be classified into three clusters with different prognoses (Figure 8F); MMS-cluster3 had the worst prognosis with the highest mRNAsi, while MMS-cluster2 had the most favorable prognosis with the lowest mRNAsi (Figure 8G). We also investigated whether there were any differences in immune and stromal scores among the three clusters. The results showed that MMS-cluster2 had higher stromal and immune scores, while MMS-cluster3 had lower stromal and immune scores (Figures 8E,H). In total, these analyses indicated that the 34 MMS-related signature could guide molecular classifications, by which the MMS clusters possessed different immune infiltration and had different prognostic outcomes.

### Development and Validation of a Nomogram

**FIGURE 9:**
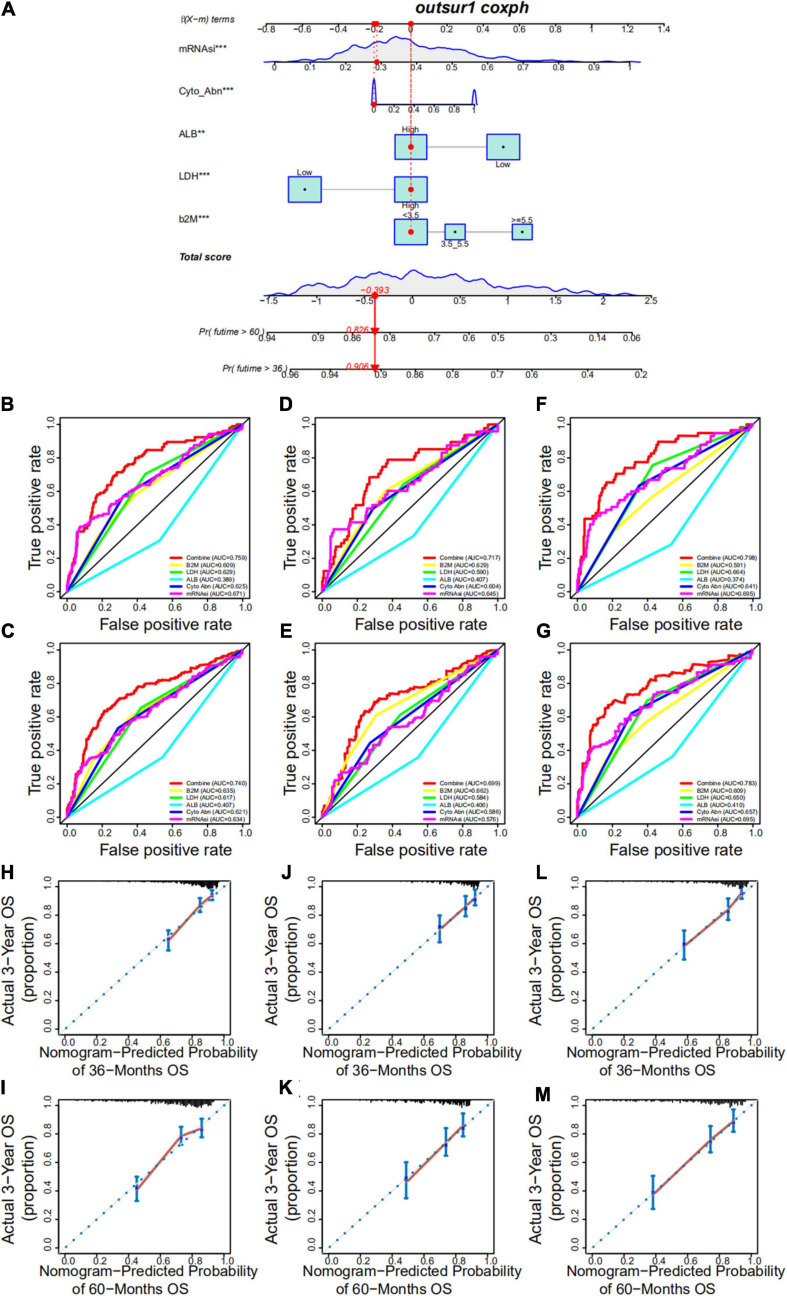
Development and validation of a nomogram. **(A)** A nomogram combining mRNAsi and other clinicopathologic covariates. **(B,C)** Receiver operating characteristic (ROC) curve to evaluate the accuracy of the 3-year **(B)** and 5-year **(C)** OS nomogram in the training cohort. **(D,E)** ROC curve to evaluate the accuracy of the 3-year **(D)** and 5-year **(E)** OS nomogram in test set 1. **(F,G)** ROC curve to evaluate the accuracy of the 3-year **(F)** and 5-year **(G)** OS nomogram in test set 2. **(H,I)** Calibration plots indicating that nomogram-predicted 3- **(H)** and 5-year **(I)** survival probabilities of the training set corresponded closely to the observed proportions. **(J,K)** Calibration plots indicating that nomogram-predicted 3- **(J)** and 5-year **(K)** survival probabilities of test set 1 corresponded closely to the observed proportions. **(L,M)** Calibration plots indicating that nomogram-predicted 3- **(L)** and 5-year **(M)** survival probabilities of test set 2 corresponded closely to the observed proportions.

Based on the multivariate Cox regression analysis, a nomogram was built to determine MM prognosis (Figure 9A), and a time-dependent ROC curve was used to evaluate the effect of the nomogram. As the data with complete clinical information was limited, we used the GSE24080 cohort as the whole dataset (training set) to construct the nomogram. To verify our nomogram, we set the random seed to 123 and used the *caret* R package to randomly cut the whole set and divided the samples into two different data sets (50%/50%) to verify the nomogram. In the whole dataset, the area under the curve was 0.759 and 0.740 for the 3- and 5-year survival, respectively (Figures 9B,C). The performance of the nomogram was far superior to that of mRNAsi, cytogenetic abnormalities, ALB, B2M, and LDH alone for assessing patient prognosis (Figures 9B,C). In addition, the nomogram showed good prediction performance in the verification cohorts, and the 3- and 5-year ROC curves of test sets 1 and 2, respectively, are shown in Figures 9D–G (Figures 9D,E, test set 1; Figures 9F,G, test set 2). A calibration curve was constructed to assess the accuracy of the nomogram. As shown in Figures 9H,I (training set) and Figures 9J–M (Figures 9J,K, test set 1; Figures 9L,M, test set 2), the combined nomogram showed good performance in predicting the 3- and 5-year survival rates of patients, and the prediction probability was close to the actual observed situation.

## Discussion

Cancer stem cells are commonly considered to be responsible for tumor persistence and progression, tumor recurrence, and resistance to traditional therapies (Seguin et al., 2015; Prasetyanti and Medema, 2017; Saygin et al., 2019). We comprehensively analyzed data from large cohorts to determine the cancer stemness of MM and found that mRNAsi was tightly associated with OS and EFS, possessing good fitness and quantification of stemness in MM. In addition, low- and high-mRNAsi groups had different features, including B2M, cytogenetic abnormalities, ALB, subgroups, and immune infiltration. This evidence indicates that stemness, as a yardstick to assess the degree of gradual loss of a differentiated phenotype and gain of progenitor and stem cell-like characteristics, strongly affects the prognosis of MM. Our researches showed that mRNAsi could effectively classify patients with MM into groups with low and high risks of poor prognosis. Additionally, the proposed mRNAsi provided additional prognostic value to existing clinicopathological prognosticators for MM. Of particular importance, this is the first study to demonstrate the clinical utility of the stemness signature as a prognostic tool in patients with MM. We observed that the prognoses of patients with MM who received different therapies (TT2 or TT3) were not significantly different. Similarly, mRNAsi did not differ significantly; however, in both the TT2 and TT3 groups, patients with high mRNAsi had shorter OS than patients with low mRNAsi. This suggests that the TT3 regimen did not significantly improve the prognosis of patients compared with the TT2 regimen. This might be because the TT3 regimen failed to remove the stemness, thus failing to influence the degree of oncogenic dedifferentiation, which might provide valuable insights for targeted therapies aimed at tumor differentiation of MM. Moreover, the stemness index-related modules and genes were identified using WGCNA and differential expression analysis. A total of 127 hub prognostic genes were found to be most significantly associated with stemness index. Pathway and process enrichment analysis revealed that these hub prognostic genes were involved in various biological functions related to the initiation and progression of MM, including the cell cycle, cell differentiation, and DNA replication and repair. Interestingly, these hub prognostic genes allowed for the discovery of innovative targets and possible targeted therapies aimed at tumor differentiation. Based on the MCODE algorithm, the most important connected network components were identified from these hub prognostic genes. We considered tumor heterogeneity and classified the MM cohorts into three MMS clusters based on the 34 stemness-related signatures. We observed distinct survival outcomes across the three MMS clusters and compared the differential mRNAsi among the three MMS clusters. In particular, MMS-cluster3 samples possessed the highest mRNAsi and had the worst OS outcomes compared with other clusters. We also applied the ESTIMATE method to evaluate the level of immune infiltrating cells in the TME of MM and found different infiltration patterns across the three clusters. These findings revealed the patterns of intra-tumor molecular heterogeneity and the different patterns of TME infiltration within MM and supported the negative regulation of the stemness index and anticancer immunity. Lastly, by integrating mRNAsi and other clinical characteristics, we proposed a prognostic nomogram that allows for individualized estimations of the 3- and 5-year OS probabilities among MM patients.

Stemness signatures have been identified in many malignancies and have different prognostic values in gastric cancer, acute myeloid leukemia, prostate cancer, and breast cancer (Ng et al., 2016; Malta et al., 2018; Wang et al., 2018; Miranda et al., 2019; Chang et al., 2020; Zhang et al., 2020). However, there are few studies on the stemness of MM and its prognostic value in MM patients. In our study, mRNAsi could stratify MM patients into two groups with notably different prognoses, which was convenient for risk stratification of MM in clinical practice. Integrated analyses also confirmed that mRNAsi is an independent prognostic biomarker in MM. In our screening in multiple cohorts, some of the stemness-related genes have never been reported in other previous researches that could predict the outcomes of MM, which was convenient to implement in clinical practice. BUB1B encodes a kinase involved in spindle checkpoint function. The protein is localized to the kinetochore and plays a role in the inhibition of the anaphase-promoting complex/cyclosome. An increasing body of literature has verified that aberrant expression of BUB1B is highly involved in the tumorigenesis and the development of various tumors (Wan et al., 2012; Qiu et al., 2020). A previous study revealed that BUB1B could promote MM cell proliferation through the CDC20/CCNB axis (Yang et al., 2015). MCM2, MCM3, MCM5, and MCM6, members of the minichromosome maintenance (MCM) family, are highly involved in DNA replication and are vital in limiting replication in the cell cycle (Freeman et al., 1999; Forsburg, 2004). Some investigations have shown that the expression of MCM family plays an important role in the prognosis of MM, and MCM2 is an independent risk factor for MM (Quan et al., 2020). Additionally, MCM2 is associated with many types of cancer, including acute lymphocytic leukemia, gallbladder cancer, and glioma (Hua et al., 2014; Liu et al., 2016; Li et al., 2018). Trichostatin A, a classical histone deacetylase inhibitor, could downregulate the expression of MCM2, and the silencing of MCM2 in colon cancer cells could induce cell cycle arrest and apoptosis as reported in a previous study (Liu et al., 2013). Therefore, MCM2 could be a potential therapeutic target for the treatment of MM. ZWILCH kinetochore protein is an essential part of the Rod–Zw10–Zwilch complex and is important in maintaining the normal function of mitotic checkpoints (Karess, 2005; Gama et al., 2017). The abnormal function of mitotic checkpoints is related to the appearance of chromosomal instability, a consensus sign of many human cancers. In MM, chromosomal instability contributes to the acquisition of tumor heterogeneity and thereby to drug resistance, disease progression, and eventual treatment failure (Fujibayashi et al., 2020; Neuse et al., 2020). Aberrant BUB1 overexpression promotes mitotic segregation errors and chromosomal instability in MM (Fujibayashi et al., 2020), and the synergistic mechanism of BUB1B and ZWILCH in the occurrence and development of MM requires further investigation.

Moreover, we proposed prognostic nomogram that contribute to individualized evaluations of the 3- and 5-year OS probabilities among patients with MM. Taken together, mRNAsi and the associated nomogram might serve as a clinically helpful tool to improve surveillance and guide decision-making regarding the administration of adjuvant chemotherapy.

Collectively, mRNAsi could effectively classify patients with MM into groups with different risks of outcomes, thereby raising the possibility that stemness might be supplementary to the conventional clinicopathological risk factors as a prognostic scheme. The 34-gene based MMS-related signature could be a good molecular classifier for uncovering distinct stemness clusters. Additionally, the proposed nomogram incorporating mRNAsi and existing clinical prognosticators might facilitate personalized surveillance and management of patients with MM.

## Data Availability Statement

The original contributions presented in the study are included in the article/Supplementary Material, further inquiries can be directed to the corresponding author/s.

## Author Contributions

JH designed the study, interpreted the data, and revised the manuscript. CB and FY performed the statistical analysis, interpreted the data, and wrote the manuscript. MW, QL, and JW provided the bioinformatic data and contributed to the manuscript revision. LC, LTL, DX, and LL provided the key scientific insights and contributed to the manuscript revision. All authors have read and approved the final version of the manuscript.

## Conflict of Interest

The authors declare that the research was conducted in the absence of any commercial or financial relationships that could be construed as a potential conflict of interest.

## Publisher’s Note

All claims expressed in this article are solely those of the authors and do not necessarily represent those of their affiliated organizations, or those of the publisher, the editors and the reviewers. Any product that may be evaluated in this article, or claim that may be made by its manufacturer, is not guaranteed or endorsed by the publisher.

## Footnotes

1
https://www.ncbi.nlm.nih.gov/geo/

2
https://metascape.org/gp/index.html#/main/step1

## References

[B1] Bader G. D. Hogue C. W. (2003). An automated method for finding molecular complexes in large protein interaction networks. *BMC Bioinform.* 4:2. 10.1186/1471-2105-4-2 12525261 PMC149346

[B2] Bataille R. Harousseau J. L. (1997). Multiple myeloma. *N. Engl. J. Med.* 336 1657–1664. 10.1056/nejm199706053362307 9171069

[B3] Chang W. Wang H. Kim W. Liu Y. Deng H. Liu H. (2020). Hormonal suppression of stem cells inhibits symmetric cell division and gastric tumorigenesis. *Cell Stem Cell* 26 739–54.e738. 10.1016/j.stem.2020.01.020 32142681 PMC7214188

[B4] Dhakal B. Girnius S. Hari P. (2016). Recent advances in understanding multiple myeloma. *F1000Res* 5:F1000FacultyRev–2053. 10.12688/f1000research.8777.1 27610224 PMC4995679

[B5] Driscoll J. J. Pelluru D. Lefkimmiatis K. Fulciniti M. Prabhala R. H. Greipp P. R. (2010). The sumoylation pathway is dysregulated in multiple myeloma and is associated with adverse patient outcome. *Blood* 115 2827–2834. 10.1182/blood-2009-03-211045 19965618 PMC2854429

[B6] Forsburg S. L. (2004). Eukaryotic MCM proteins: beyond replication initiation. *Microbiol. Mol. Biol. Rev.* 68 109–131. 10.1128/mmbr.68.1.109-131.2004 15007098 PMC362110

[B7] Freeman A. Morris L. S. Mills A. D. Stoeber K. Laskey R. A. Williams G. H. (1999). Minichromosome maintenance proteins as biological markers of dysplasia and malignancy. *Clin. Cancer Res.* 5 2121–2132. 10473096

[B8] Fujibayashi Y. Isa R. Nishiyama D. Sakamoto-Inada N. Kawasumi N. Yamaguchi J. (2020). Aberrant BUB1 overexpression promotes mitotic segregation errors and chromosomal instability in multiple myeloma. *Cancers* 12:2206. 10.3390/cancers12082206 32781708 PMC7464435

[B9] Gama J. B. Pereira C. Simões P. A. Celestino R. Reis R. M. Barbosa D. J. (2017). Molecular mechanism of dynein recruitment to kinetochores by the Rod-Zw10-Zwilch complex and Spindly. *J. Cell Biol.* 216 943–960. 10.1083/jcb.201610108 28320824 PMC5379953

[B10] Hua C. Zhao G. Li Y. Bie L. (2014). Minichromosome maintenance (MCM) family as potential diagnostic and prognostic tumor markers for human gliomas. *BMC Cancer* 14:526. 10.1186/1471-2407-14-526 25046975 PMC4223428

[B11] Karess R. (2005). Rod-Zw10-Zwilch: a key player in the spindle checkpoint. *Trends Cell Biol.* 15 386–392. 10.1016/j.tcb.2005.05.003 15922598

[B12] Langfelder P. Horvath S. (2008). WGCNA: an R package for weighted correlation network analysis. *BMC Bioinform.* 9:559. 10.1186/1471-2105-9-559 19114008 PMC2631488

[B13] Lapidot T. Sirard C. Vormoor J. Murdoch B. Hoang T. Caceres-Cortes J. (1994). A cell initiating human acute myeloid leukaemia after transplantation into SCID mice. *Nature* 367 645–648. 10.1038/367645a0 7509044

[B14] Li S. Wang C. Wang W. Liu W. Zhang G. (2018). Abnormally high expression of POLD1, MCM2, and PLK4 promotes relapse of acute lymphoblastic leukemia. *Medicine* 97:e10734. 10.1097/md.0000000000010734 29768346 PMC5976347

[B15] Lian H. Han Y. P. Zhang Y. C. Zhao Y. Yan S. Li Q. F. (2019). Integrative analysis of gene expression and DNA methylation through one-class logistic regression machine learning identifies stemness features in medulloblastoma. *Mol. Oncol.* 13 2227–2245. 10.1002/1878-0261.12557 31385424 PMC6763787

[B16] Liu Y. He G. Wang Y. Guan X. Pang X. Zhang B. (2013). MCM-2 is a therapeutic target of Trichostatin A in colon cancer cells. *Toxicol. Lett.* 221 23–30. 10.1016/j.toxlet.2013.05.643 23770000

[B17] Liu Z. Yang Z. Jiang S. Zou Q. Yuan Y. Li J. (2016). MCM2 and TIP30 are prognostic markers in squamous cell/adenosquamous carcinoma and adenocarcinoma of the gallbladder. *Mol. Med. Rep.* 14 4581–4592. 10.3892/mmr.2016.5851 27748889 PMC5102005

[B18] Malta T. M. Sokolov A. Gentles A. J. Burzykowski T. Poisson L. Weinstein J. N. (2018). Machine learning identifies stemness features associated with oncogenic dedifferentiation. *Cell* 173:338–354.e315. 10.1016/j.cell.2018.03.034 29625051 PMC5902191

[B19] Martinez-Lopez J. Blade J. Mateos M. V. Grande C. Alegre A. García-Laraña J. (2011). Long-term prognostic significance of response in multiple myeloma after stem cell transplantation. *Blood* 118 529–534. 10.1182/blood-2011-01-332320 21482708

[B20] Matsui W. Wang Q. Barber J. P. Brennan S. Smith B. D. Borrello I. (2008). Clonogenic multiple myeloma progenitors, stem cell properties, and drug resistance. *Cancer Res.* 68 190–197. 10.1158/0008-5472.Can-07-3096 18172311 PMC2603142

[B21] Miranda A. Hamilton P. T. Zhang A. W. Pattnaik S. Becht E. Mezheyeuski A. (2019). Cancer stemness, intratumoral heterogeneity, and immune response across cancers. *Proc. Natl. Acad. Sci. U.S.A.* 116 9020–9029. 10.1073/pnas.1818210116 30996127 PMC6500180

[B22] Neuse C. J. Lomas O. C. Schliemann C. Shen Y. J. Manier S. Bustoros M. (2020). Genome instability in multiple myeloma. *Leukemia* 34 2887–2897. 10.1038/s41375-020-0921-y 32651540

[B23] Ng S. W. Mitchell A. Kennedy J. A. Chen W. C. McLeod J. Ibrahimova N. (2016). A 17-gene stemness score for rapid determination of risk in acute leukaemia. *Nature* 540 433–437. 10.1038/nature20598 27926740

[B24] Nowak D. Stewart D. Koeffler H. P. (2009). Differentiation therapy of leukemia: 3 decades of development. *Blood* 113 3655–3665. 10.1182/blood-2009-01-198911 19221035 PMC2943835

[B25] O’Brien C. A. Pollett A. Gallinger S. Dick J. E. (2007). A human colon cancer cell capable of initiating tumour growth in immunodeficient mice. *Nature* 445 106–110. 10.1038/nature05372 17122772

[B26] Prasetyanti P. R. Medema J. P. (2017). Intra-tumor heterogeneity from a cancer stem cell perspective. *Mol. Cancer* 16:41. 10.1186/s12943-017-0600-4 28209166 PMC5314464

[B27] Qiu J. Zhang S. Wang P. Wang H. Sha B. Peng H. (2020). BUB1B promotes hepatocellular carcinoma progression via activation of the mTORC1 signaling pathway. *Cancer Med.* 9 8159–8172. 10.1002/cam4.3411 32977361 PMC7643650

[B28] Quan L. Qian T. Cui L. Liu Y. Fu L. Si C. (2020). Prognostic role of minichromosome maintenance family in multiple myeloma. *Cancer Gene Ther.* 27 819–829. 10.1038/s41417-020-0162-2 31959909

[B29] Ritchie M. E. Phipson B. Wu D. Hu Y. Law C. W. Shi W. (2015). limma powers differential expression analyses for RNA-sequencing and microarray studies. *Nucleic Acids Res.* 43:e47. 10.1093/nar/gkv007 25605792 PMC4402510

[B30] Röllig C. Knop S. Bornhäuser M. (2015). Multiple myeloma. *Lancet* 385 2197–2208. 10.1016/s0140-6736(14)60493-1 25540889

[B31] Saygin C. Matei D. Majeti R. Reizes O. Lathia J. D. (2019). Targeting cancer stemness in the clinic: from hype to hope. *Cell Stem Cell* 24 25–40. 10.1016/j.stem.2018.11.017 30595497

[B32] Seguin L. Desgrosellier J. S. Weis S. M. Cheresh D. A. (2015). Integrins and cancer: regulators of cancer stemness, metastasis, and drug resistance. *Trends Cell Biol.* 25 234–240. 10.1016/j.tcb.2014.12.006 25572304 PMC4380531

[B33] Shi L. Campbell G. Jones W. D. Campagne F. Wen Z. Walker S. J. (2010). The MicroArray Quality Control (MAQC)-II study of common practices for the development and validation of microarray-based predictive models. *Nat. Biotechnol.* 28 827–838. 10.1038/nbt.1665 20676074 PMC3315840

[B34] Singh S. K. Hawkins C. Clarke I. D. Squire J. A. Bayani J. Hide T. (2004). Identification of human brain tumour initiating cells. *Nature* 432 396–401. 10.1038/nature03128 15549107

[B35] Sokolov A. Paull E. O. Stuart J. M. (2016). ONE-CLASS DETECTION OF CELL STATES IN TUMOR SUBTYPES. *Pac. Symp. Biocomput.* 21 405–416. 26776204 PMC4856035

[B36] Wan X. Yeung C. Kim S. Y. Dolan J. G. Ngo V. N. Burkett S. (2012). Identification of FoxM1/Bub1b signaling pathway as a required component for growth and survival of rhabdomyosarcoma. *Cancer Res.* 72 5889–5899. 10.1158/0008-5472.Can-12-1991 23002205 PMC3500453

[B37] Wang T. Fahrmann J. F. Lee H. Li Y. J. Tripathi S. C. Yue C. (2018). JAK/STAT3-regulated fatty acid β-oxidation is critical for breast cancer stem cell self-renewal and chemoresistance. *Cell Metab.* 27 136–150.e135. 10.1016/j.cmet.2017.11.001 29249690 PMC5777338

[B38] Wilkerson M. D. Hayes D. N. (2010). ConsensusClusterPlus: a class discovery tool with confidence assessments and item tracking. *Bioinformatics* 26 1572–1573. 10.1093/bioinformatics/btq170 20427518 PMC2881355

[B39] Yang Y. Gu C. Luo C. Li F. Wang M. (2015). BUB1B promotes multiple myeloma cell proliferation through CDC20/CCNB axis. *Med. Oncol.* 32:81. 10.1007/s12032-015-0542-x 25698537

[B40] Yoshihara K. Shahmoradgoli M. Martínez E. Vegesna R. Kim H. Torres-Garcia W. (2013). Inferring tumour purity and stromal and immune cell admixture from expression data. *Nat. Commun.* 4:2612. 10.1038/ncomms3612 24113773 PMC3826632

[B41] Zhan F. Huang Y. Colla S. Stewart J. P. Hanamura I. Gupta S. (2006). The molecular classification of multiple myeloma. *Blood* 108 2020–2028. 10.1182/blood-2005-11-013458 16728703 PMC1895543

[B42] Zhang C. Chen T. Li Z. Liu A. Xu Y. Gao Y. (2020). Depiction of tumor stemlike features and underlying relationships with hazard immune infiltrations based on large prostate cancer cohorts. *Brief Bioinform.* 22:bbaa211. 10.1093/bib/bbaa211 32856039

[B43] Zhou Y. Zhou B. Pache L. Chang M. Khodabakhshi A. H. Tanaseichuk O. (2019). Metascape provides a biologist-oriented resource for the analysis of systems-level datasets. *Nat. Commun.* 10:1523. 30944313 10.1038/s41467-019-09234-6 PMC6447622

